# The complete chloroplast genome of *Impatiens uliginosa* Franch., an endemic species in Southwest China

**DOI:** 10.1080/23802359.2019.1687024

**Published:** 2019-11-06

**Authors:** Chao Luo, Wulue Huang, Jiapeng Zhu, Zhixi Feng, Yingli Liu, Yang Li, Xinyi Li, Haiquan Huang, Meijuan Huang

**Affiliations:** a College of Landscape Architecture, Southwest Forestry University, Kunming, China;; b Research and Development Center of Landscape Plants and Horticulture Flowers, Southwest Forestry University, Kunming, China

**Keywords:** *Impatiens uliginosa* Franch., endemic species, chloroplast genome

## Abstract

The complete chloroplast genome sequence of *Impatiens uliginosa* Franch., an endemic species in Southwest China, we research genetic and phylogenetic relationship with other species in an effort to provide genomic resources useful for promoting its conservation and utilization. The total chloroplast genome size of *I. uliginosa* is 152,609 bp, with a typical quadripartite structure including a pair of inverted repeat (IRs, 25,871 bp) regions separated by a small single copy (SSC, 17,502 bp) region and a large single copy (LSC, 83,365 bp) region. The overall GC content of *I. uliginosa* plastid genome was 36.8%. The whole chloroplast genome contains 136 genes, including 89 protein-coding genes (PCGs), 38 transfer RNA genes (tRNAs), and 8 ribosomal RNA genes (rRNAs). Among these genes, 15 genes have one intron and 2 genes contain two introns. To investigate the evolution status, the phylogenetic tree based on APG III from 12 complete chloroplast plastomes of Ericales supports close relationships. According to the phylogenetic topologies, *I. uliginosa* was closely related to *I. piufanensis.*

*Impatiens*, one of the three most widely used bedding flowers in the world, is known for its rich corolla colors and ornamental values. In addition, it has some medicinal and cosmetic values because of containing many bioactive compounds including 2-methoxy-1,4-naphthoquinone, quercetin, and lawsone (Fischer 2004; Yu et al. 2016). The largest angiosperm genus *Impatiens* includes over 1000 species distribute in the world, with 250 species in China (Grey-Wilson 1980). *Impatiens* is famous for its taxonomic difficulty. Nevertheless, to date, a generally accepted worldwide infrageneric classification for *Impatiens* is lacking (Janssens et al. 2012). *Impatiens uliginosa* Franch., an endemic annual and perennial species in China, occurs only at high altitudes range from 1600 to 2300 m of Yunnan, Guizhou, and Guangxi in the southwest of China. It has not only high ornamental values and its own characteristics including various flower colors and flower diameters, unique flower shapes, etc.(Yu et al. 2016), but also good adaptability and high resistance in the biotic and abiotic stresses (Chen 2001). Meanwhile, all year round it blooms in Dian Pond of Kunming in Yunnan. For a better understanding of *I. uliginosa* Franch*.,* it is essential to reconstruct a phylogenetic tree of the *Impatiens* species based on high-throughput sequencing approaches.

The fresh leaves of *I. uliginosa* were sampled from Dian Pond of Kunming (Yunnan, China; coordinates: 102°75′89″E, 25°06′03″N; altitude: 1952.4 m). Total genomic DNA was extracted with the BioTeke Plant Genomic DNA Preps Kit (BioTeke Corporation, Beijing, China). The voucher specimens of *I. uliginosa* samples were properly deposited at the herbarium of Southwest Forestry University, which specimen Accession number is SWFU-IBDSJF20180810, and DNA samples were properly stored in College of Landscape Architecture and Research and Development Center of Landscape Plants and Horticulture Flowers, Southwest Forestry University, Kunming, Yunnan, China. Total genomic DNA was used to generate libraries with an average insert size of 400 bp and sequences using the Illumina Hiseq X platform. Approximately, 1.95 GB of raw data was generated with 150 bp paired-end read lengths. The complete chloroplast genome used the software of GetOrganelle by the raw data with *I. pinfanensis* (GenBank_MG162586.1) as the reference (Jin et al. 2018). Genome annotation was performed with the program Geneious R10 (Biomatters Ltd., Auckland, New Zealand). The cpDNA sequence with complete annotation information was deposited at GenBank database under the accession number MN533984.

The plastome of *I. uliginosa* is a double-stranded circular DNA with a length of 152,609 bp, containing a pair of inverted repeats (IRs) of 25,871 bp, a large single copy (LSC) region of 83,365 bp, and a small single copy (SSC) region of 17,502 bp. The overall GC content of the whole plastome was 36.8%, while the corresponding values of the LSC, SSC, and IR regions were 34.5, 29.6, and 42.9%, respectively. The complete chloroplast plastome annotated 136 genes, including 89 protein-coding genes (PCGs), 38 tRNA genes, and 8 rRNA genes. Among these genes, there were 15 genes (*rps16*, *rps12*, *rpoC1*, *rpl2*, *petB*, *ndhB*, *ndhA*, *atpF*, *trnK-UUU*, *trnG-GCC*, *trnL-UAA*, *trnV-UAC*, *trnI-GAU*, *trnA-UGC,* and *trnI-GAU*) with one intron and two genes (*ycf3* and *clpP*) with two introns.

To determine the phylogenetic location of *I. uliginosa* with respect to the other impatiens with fully sequenced chloroplast genomes. With the plastome of *Sinojackia xylocarpa* (MH782178.1) in the family of Sinojackia as out-group, 13 chloroplast genome sequences of Ericales were aligned by the MAFFT software, version 7.0 (Katoh and Standley 2013). A maximum-likelihood method for phylogenetic analysis was performed based on GTR + I+G model in the RAxML program, version 8.0 with 1000 bootstrap replicates (Darriba et al. 2012; Stamatakis 2014). Our chloroplast phylogenomic analysis of the Balsaminaceae, in agreement with previously published phylogenetic studies (Li et al. 2018), reveals that there are close relationships between *I. uliginosa*, *I. piufanensis* (MG162586.1), and *H. triflora* (MG162585.1) (Figure 1).

**Figure 1.**
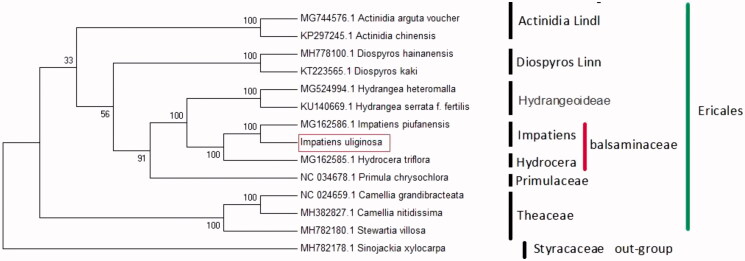
The ML phylogenetic tree for *Impatiens uliginosa* based on 12 chloroplast genome sequences of Ericales and one plastome of *Sinojackia xylocarpa* (MH782178.1) as outgroup. Numbers on the nodes are bootstrap values from 1000 replicates. Accession numbers: *Camellia grandibracteata* (NC_024659.1), *Camellia nitidissima* (MH382827.1), *Stewartia villosa* (MH782180.1), *Diospyros hainanensis* (MH778100.1), *Diospyros kaki* (KT223565.1), *Actinidia argute* (MG744576.1), *Actinidia chinensis* (KP297245), *Primula chrysochlora* (NC_034678.1), *Hydrangea heteromalla* (MG524994.1), *Hydrangea serrata* (KU140669.1), *Hydrocera triflora* (MG162585.1), and *I. piufanensis* (MG162586.1).
